# Comprehensive Evaluation of Personal, Clinical, and Radiation Dosimetric Parameters for Acute Skin Reaction during Whole Breast Radiotherapy

**DOI:** 10.1155/2016/3656574

**Published:** 2016-08-07

**Authors:** Dae Sik Yang, Jung Ae Lee, Won Sup Yoon, Nam Kwon Lee, Young Je Park, Suk Lee, Chul Yong Kim, Gil Soo Son

**Affiliations:** ^1^Department of Radiation Oncology, Guro Hospital, Korea University Medical Center, 148 Gurodong-ro, Guro-gu, Seoul 08308, Republic of Korea; ^2^Department of Radiation Oncology, Ansan Hospital, Korea University Medical Center, 123 Jeokguem-ro, Ansan, Gyeonggi 15355, Republic of Korea; ^3^Department of Radiation Oncology, Anam Hospital, Korea University Medical Center, 73 Inchon-ro, Seongbuk-gu, Seoul 02841, Republic of Korea; ^4^Department of Surgery, Ansan Hospital, Korea University Medical Center, 123 Jeokguem-ro, Ansan, Gyeonggi 15355, Republic of Korea

## Abstract

Skin reaction is major problem during whole breast radiotherapy. To identify factors related to skin reactions during whole breast radiotherapy, various personal, clinical, and radiation dosimetric parameters were evaluated. From January 2012 to December 2013, a total of 125 patients who underwent breast conserving surgery and adjuvant whole breast irradiation were retrospectively reviewed. All patients had both whole breast irradiation and boost to the tumour bed. Skin reaction was measured on the first day of boost therapy based on photography of the radiation field and medical records. For each area of axilla and inferior fold, the intensity score of erythema (score 1 to 5) and extent (score 0 to 1) were summed. The relationship of various parameters to skin reaction was evaluated using chi-square and linear regression tests. The *V*_100_ (volume receiving 100% of prescribed radiation dose, *p* < 0.001, both axilla and inferior fold) and age (*p* = 0.039 for axilla and 0.026 for inferior fold) were significant parameters in multivariate analyses. The calculated axilla dose (*p* = 0.003) and breast separation (*p* = 0.036) were also risk factors for axilla and inferior fold, respectively. Young age and large *V*_100_ are significant factors for acute skin reaction that can be simply and cost-effectively measured.

## 1. Introduction

Breast conserving surgery is an initial approach for treating early breast cancer because it preserves cosmetic appearance and reduces major surgical sequelae. Adjuvant radiotherapy after breast conserving surgery reduces local recurrence and improves overall survival by irradiating the remaining cancer cell foci [1]. Therefore, adjuvant radiotherapy after breast conserving surgery is standard treatment for early stage breast cancer.

Conventional radiotherapy to whole breast uses the opposed tangential fields with an appropriate wedge filter. Progress in techniques used to calculate radiation dose distribution and accurate delivery of the radiation beams has resulted in modified radiotherapy techniques, such as the field-in-field technique that can be applied to whole breast irradiation. Recent trials have reported that these methods reduce the occurrence of moist desquamation, changes in breast appearance, and palpable induration [2, 3].

However, some patients continue to experience severe skin reaction during radiotherapy. Skin toxicity affects quality of life [4] and increases out-of-pocket costs [5]. Skin reactions develop more severely on the lateral upper quadrants compared to other areas [6]. Various clinical factors, including body mass index, large breast size, and smoking are risk factors for skin reactions, as well as treatment-related factors including concomitant target and hormonal therapies [7–10]. Some genetic factors (e.g., polymorphisms in XRCC1, XRCC3, GSTP1, eNOS, ataxia-telangiectasia mutated gene, and the BRCA mutation) have been recently recognized as risk factors for skin reactions [11–15]. However, the cost of a gene examination is too high to apply to the general population and the relationship between genetic factors and skin reaction is weak.

When radiotherapy is planned through virtual simulation, the various dosimetric parameters are used as references to evaluate not only the coverage of target volume but also avoidance of organs at risk. Through the analyses of these parameters, the quality of radiotherapy can be improved. In our study, we evaluated various dosimetric parameters, in addition to personal and clinical parameters, to identify factors related to skin reactions.

## 2. Methods

### 2.1. Patients

In our retrospective study, patients with adjuvant whole breast irradiation including the boost after breast conserving surgery due to breast cancer from January 2012 to December 2013 had been enrolled in Ansan Hospital, Korea University Medical Center. Patients undergoing supraclavicular or axillary irradiation with another field were excluded because the overlapping fields between whole breast and axillary area can be a bias for skin reactions. Other exclusion criteria were (1) being <20 years of age and >70 years of age; (2) bolus during irradiation; (3) artificial implant in the ipsilateral breast; (4) whole breast treatment duration >40 days, except for the cause of skin toxicity; (5) bilateral breast irradiation; (6) history of other radiotherapies; and (7) concomitant primary malignancy that required adjuvant therapy. Medical records and technical radiotherapy reports were reviewed after Institutional Review Board approval of our study.

### 2.2. Radiotherapy

The dose prescriptions were identical. A total of 50 Gy divided into 25 fractions with 6 MV X-ray was delivered to the whole breast, and the tumour bed boost was continued with an intended dose of 10 Gy with 5 fractions or 15 Gy with 7 fractions. A Brilliance Big Bore Oncology computed tomography (CT) system (Philips Medical Systems, Best, the Netherlands) was utilized, and the Breastboard (Civico, Orange City, IA, USA) was used as an immobilization device. The setup was performed with the patient in the supine position with both arms elevated above the head. The CT scans were sliced with a 5 mm thickness. Varian Eclipse version 8.6.1.5 (Varian Medical Systems Inc., Palo Alto, CA, USA) was used for radiotherapy planning. The planning target volume (PTV) of the whole breast was edited 5 mm from the body surface. The source-to-surface distance method and the anisotropic analytical algorithm calculation model were applied. One to three subsegments were used in each medial and lateral beam direction to create our field-in-field technique for whole breast irradiation. In general, the radiotherapy plan of whole breast irradiation was used if it satisfied the following criteria: *V*_90_ (*V*_*X*_: a covered volume by the *X*% of prescribed dose) > 99%, *V*_95_ > 90%, maximum dose < 107%, mean breast dose ≈ 100%, and mean ipsilateral lung dose < 10 Gy. Electronic portal images were taken weekly for verification during the entire radiation period. Photographs indicating the radiation fields with therapeutic position were taken on the first day of setup for whole breast and tumour bed boost and were preserved in our radiotherapy technical records.

### 2.3. Skin Reaction Measurement

Acute skin toxicity was checked on the first day of the tumour bed boost by radiation oncologist and written down in our medical record. For this study, a photographic comparison between the initial whole breast setup and the initial boost setup and a review of medical data were retrospectively done by a 15-year experienced radiation oncologist and a 7-year experienced nurse. The breast skin reaction was measured on each axilla and inferior fold area. The intensity of the skin reaction was divided into five levels according to skin colour changes and erythema. Scores of 1, 2, 3, 4, and 5 were given for faint, mild, moderate, severe, and wet desquamation skin reactions, respectively. If radiotherapy was delayed by desquamation during whole breast irradiation, it was given a score of 5. If the score was ≥3 and the reaction was greater than palm size, a score of 1 was added to the intensity score. Therefore, the final acute skin reaction score was 1–6.

### 2.4. Parameters

The clinical/individual parameters were as follows: age (year), body mass index (kg/m^2^), laterality (right side versus left side), pT-stage (0 or 1 versus 2 or 3), pN-stage (0 versus 1), method of axillary dissection (no surgery or sentinel node dissection versus axillary node dissection), chemotherapy before adjuvant radiotherapy, and hormonal therapy during radiotherapy.

**Figure 1:**
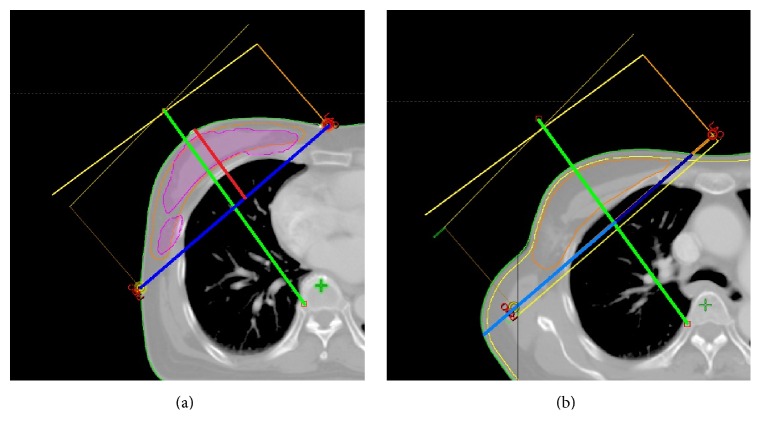
Diagram to measure various dosimetric factors. (a) Breast height (red line) means the distance between the posterior filed border and the apex of breast and breast separation (blue line) means the distance from medial to lateral radiation field border on central axis. *V*_100_ is indicated as magenta area and a perpendicular line to divide the breast separation as halves is indicated as green line. (b) Green line is the extension line from the central axis (a) and asymmetry ratio of lateral separation to medical separation on the upper border is the distance of dark blue to the distance of light blue. A virtual contour that edited 2 mm from the body was generated to measure point doses indicated as yellow line.

The dosimetric parameters were as follows: breast height (the distance between the posterior filed border and the apex of breast at the nipple axis), breast separation (the distance from medial to lateral radiation field border at the central axis), absolute volume including the PTV, *V*_100_, *V*_95_, *V*_90_, *V*_80_, and *V*_50_, distance from the lower margin of humeral head to the upper border of radiation field, field size of the *Y*-axis, asymmetry ratio of lateral separation to medical separation on the upper border (perpendicular line that halved the breast at the central axis as medial and lateral halves were extended to the upper border, and it divided off the medial and lateral separation on the upper border), and the calculated point doses on the radiotherapy planning system (Figure 1). A virtual contour that edited 2 mm from the body was generated to measure point doses, and the surface point dose was calculated. The axillary dose was measured in the axillary fold on the axis 1 cm below the top of the PTV. The inferior fold dose was measured on a vertical line from the nipple. The inner half dose was measured on the medial 5 cm from the nipple. Doses are presented as relative percentages to the prescribed dose.

### 2.5. Statistics

The statistical analysis was conducted on skin toxicity and risk factors using SPSS version 20.0 software (SPSS Inc., Chicago, IL, USA). The chi-square test (linear to linear correlation) and the Pearson correlation analyses were used to assess the relationship between skin toxicity scores and risk factors, which were presented as categorical and continuous variables, respectively. The absolute values of Pearson's correlation coefficients (*r*; *r* ≥ 0.75, 0.4 ≤ *r* < 0.75, and *r* < 0.4) were defined as strong, moderate, and weak relations, respectively. For various volumetric factors including the PTV, *V*_100_, *V*_95_, *V*_90_, *V*_80_, and *V*_50_, the correlation between them was checked by Pearson's correlation analyses and if these factors have the strong relations, a representative parameter with the strongest significance *p* value was used as a volumetric factor. Risk factors with a *p* value < 0.10 in a simple regression model were entered into the multiple regression analyses using the backward elimination method. The *p* values < 0.05 were considered significant.

## 3. Results

### 3.1. Patient Characteristics

A total of 125 patients among 134 who received whole breast irradiation with a tumour bed boost were enrolled. The causes of exclusion were age >70 years (two patients), use of a bolus (one patient), breast implant (one patient), and long treatment period for whole breast irradiation without skin desquamation (five patients). Median age was 47 years (range, 28–70 years). Seven patients had ductal carcinoma in situ and others had malignancies. Of the 78 patients who underwent chemotherapy, 17 and 47 patients received doxorubicin-based and docetaxel-based chemotherapy, respectively. The intervals from operation to the first day of radiotherapy and from the first fraction of whole breast therapy to the first fraction of the tumour bed boost were a median of 111 days (range, 26–219 days) and a median of 37 days (range 35–62 days), respectively (Table 1).

### 3.2. Intensity of Skin Reaction

Two patients had their treatment interrupted due to skin desquamation. Scores of 1-2, 3-4, and 5-6 were received for axilla skin reactions in 26.4%, 40.8%, and 32.8% of patients, respectively, and 44.8%, 25.6%, and 29.6% presented for inferior fold skin reactions, respectively (Table 2). The axilla and inferior fold skin reaction scores were correlated (Pearson's correlation coefficient [*r*], 0.848; *p* < 0.001).

### 3.3. Univariate Analyses for Skin Reaction

No parameters including laterality, T-stage, N-stage, extent of axillary dissection, chemotherapy, or hormonal therapy were significant for acute skin reaction; however, the young age group (≤50 years) had a tendency to have more severe skin reactions than those in the old age group (*p* = 0.088 for axilla and *p* = 0.012 for inferior fold) (Table 3).

Because the parameters for the various volumes were strongly correlated (*r* > 0.950) and among these parameters *V*_100_ was significant in the regression, we used *V*_100_ in this study. In univariate analyses for both axilla and inferior fold skin reactions, body mass index (*p* = 0.001 and *p* = 0.005, resp.), breast height (both *p* < 0.001), *V*_100_ (both *p* < 0.001), and breast separation at the central axis (*p* = 0.002 and *p* = 0.016, resp.) were significant. The calculated axilla point dose was related to axilla skin reactions (*p* < 0.001), whereas the inferior fold dose was not related to inferior fold skin reactions. However, the inferior fold skin reaction was marginally associated with the inner half dose (*p* = 0.074) (Table 4).

### 3.4. Multivariate Analyses for Skin Reaction

A multivariate analysis was conducted with the risk parameters from the univariate analysis and with age, which was significant on a chi-square test. Age (*p* = 0.039), *V*_100_ (*p* < 0.001), and the calculated axilla dose (*p* = 0.033) were risk factors for the axilla skin reactions, with an *r* value of 0.463 (*p* < 0.001). Age (*p* = 0.026), *V*_100_ (*p* < 0.001), and breast separation (*p* = 0.036) were risk factors for the inferior fold skin reactions, with an *r* value of 0.465 (*p* < 0.001).

**Figure 2:**
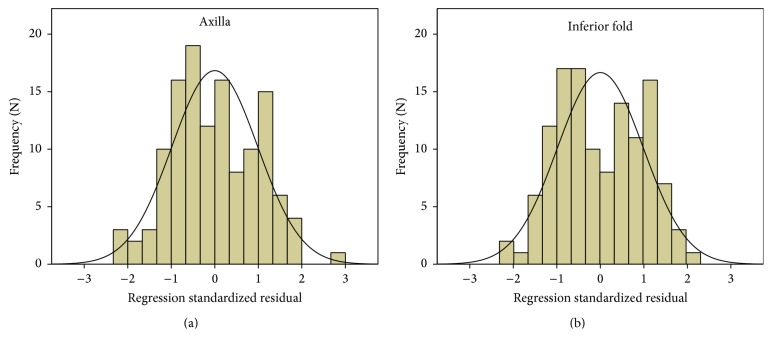
Histograms of the residual between the predictability model and observability. (a) Axilla. (b) Inferior fold.

The residuals between the predictability model and observability were a mean value of 0 and a standard deviation of 0.99 for both the axilla (range. −2.26–2.88) and inferior fold (range, −2.29–2.12). The range of predicted score ±1 covered 64.8% (81/125) and 61.1% (77/125) of the observed scores for the axilla and inferior fold areas, respectively (Figure 2).

## 4. Discussion

We performed the conventional fractionated whole breast radiotherapy in 125 consecutive patients to evaluate breast skin toxicity. Among various parameters including individual/clinical and radiotherapy dosimetric characteristics, younger age and a higher *V*_100_ were related to severe acute skin reactions, as higher calculated point dose on the radiotherapy planning system and shorter breast separation were also related to axilla and inferior fold skin reactions, respectively. Using these parameters, we proposed predictive models for skin reactions in the axilla and inferior fold areas. The Radiation Therapy Oncology Group (RTOG) scale that divides skin reactions from grade 0 to grade 4 is a general method [16]. However, a grade 4 on the RTOG scale is rarely presented in a modern radiation technique, and the range between grades 1 and 2 on the RTOG scale had a marked difference of skin colour change. Therefore, we revised the RTOG scale, and grades 0, 1-2, and 3-4 on the RTOG scale were correlated with our intensity scores of 1, 2–4, and 5, respectively. In addition, we investigated the extent of skin reactions. Some studies have used corneometry for skin dryness and colourimetry for skin erythema as objective examinations of skin toxicity [17, 18]. Another study used a patient-reported questionnaire for outcome measurements [19]. Despite the limitations of a photo documentation method, our patients were Asians with light peach skin colour. Therefore, it was relatively easy to discriminate the intensity of skin erythema, which might decrease interobserver variability.

Body mass index, breast height, and the volumetric factors (PTV, *V*_100_, *V*_95_, *V*_90_, *V*_80_, and *V*_50_) were generally associated with obesity and breast size. Among the volumetric factors, *V*_100_ was the most significant parameter that explained the most objective high dose irradiated volume because the PTV had interobserver variation and others included broader areas with lower doses. In addition, the *V*_100_ represents specific three-dimensional breast size better than body mass index or breast height. Therefore, *V*_100_ was the most powerful factor related to skin toxicity in the multivariate analysis. Another report examining breast skin toxicity suggested that *V*_107_ within the PTV and *V*_110_ within the treated volume are risk factors [20]. Because of our planning principle to reduce the maximum dose to within 107%, only one patient violated this principle, and *V*_107_ was not evaluated in our study. One advantage of the field-in-field technique is that it reduced the hot irradiated area [3]. In another study, the *V*_50_ is associated with cosmetic outcome after accelerated partial breast irradiation [21].

In our study, median age was 47 years, and 53 patients were ≤45 years. Although continuous age was not significant in the univariate analysis, some associations were observed in ordinal variables for age; therefore, age was entered in the multivariate analysis. In contrast, a Western study reported that postmenopausal status is a risk factor, although patients undergoing mastectomy were eligible in that study and both the age distribution and skin reaction endpoints were different from those in our study [9].

The calculated radiation dose on the planning system was a focus of our study. The inner quadrant dose was lower than the axilla and inferior fold doses at similar depths, and skin reactions in the axilla and inferior fold were more prominent than those in the inner quadrant. However, the axilla dose was related to skin toxicity, whereas the inferior fold dose had no association with skin toxicity. Because some inferior fold cases had a steep gradient and the calculated dose was the point dose, the calculated dose for the inferior fold may have incorrectly represented the inferior fold area. Our measured depth for the calculated point dose on the planning system was approximately 2 mm. A study that examined skin thickness by ultrasound after a median of 20.5 months of adjuvant radiotherapy showed that skin thicknesses of the irradiated and contralateral healthy breast were 2.13 ± 0.72 mm and 1.61 ± 0.29 mm, respectively [22].

The *r* values in the multivariate analysis were 0.463 for the axilla and 0.465 for the inferior fold, possibly because some factors, such as smoking history, photosensitivity history, and genetic factors, were not measured in our study. We evaluated acute skin reaction cross-sectionally. We limited the time of whole breast irradiation and evaluated toxicity just before boost therapy to reduce the bias of longer treatment time and additional dose. However, our study had some limitations, as the endpoint was not the peak time of skin reactions and no evaluation of late toxicity was conducted. Our results should be interpreted carefully because our cohort was only northeastern Asians, who have a smaller breast size and body mass index than those of Western populations.

## 5. Conclusion

In conclusion, we evaluated parameters related to skin toxicity after adjuvant whole breast irradiation in a northeastern Asian cohort. Our results suggested that age and *V*_100_ are cost-effective and easily measurable parameters for acute skin reaction during whole breast radiotherapy. Because modern radiotherapy, such as the field-in-field technique, decreases radiotoxicity by reducing the hot-spot, we thought that only a few patients should require the use of skin protective drugs or topical agents. Our skin toxicity results could be useful for defining patients who are susceptible to skin toxicity and for successfully applying an effective protective drug.

## Figures and Tables

**Table 1:** Patient characteristics.

Parameters	Median (range) or *N* : *N*
Age (years)	47 (28–70)
Laterality (right : left)	67 : 58
Body mass index (kg/m^2^)	23.4 (17.8–37.9)
T-stage (Tis, T0 : T2)	91 : 34
N-stage (N0 : N1)	106 : 19
Method of axillary dissection (no, SLND^†^ : ALND^‡^)	89 : 36
Chemotherapy (no : yes)	47 : 78
Hormonal therapy (no : yes)	32 : 93
Time interval from surgery to radiotherapy (days)	111 (26–219)
Period of whole breast irradiation (days)	36 (33–46)
Breast volume (mL)	
Planning target volume	499 (179–1444)
*V*_100_^§^	351 (154–909)
*V*_95_^§^	708 (310–1732)
*V*_90_^§^	882 (401–2184)
*V*_80_^§^	1050 (494–2547)
*V*_50_^§^	1319 (653–2973)
Breast height (cm)	3.4 (1.6–6.5)
Breast separation (cm)	19.8 (15.1–26.7)
Calculated dose/prescribed dose (%)	
Axilla	92.4 (71.2–99.3)
Inferior fold	95.8 (80.8–101.5)
Inner half	76.2 (51.9–89.1)
*Y*-field size (cm)	17.5 (15.5–20.5)
Distance from distal humerus head to upper border (cm)	1.3 (0–4.3)
Ratio of lateral to medial separation on upper border	1.62 (1.06–2.31)

^†^SLND: sentinel lymph node dissection; ^‡^ALND: axillary lymph node dissection; ^§^ *V*_*X*_: a covered volume by the *X*% of prescribed dose.

**Table 2:** The extent of skin reaction during adjuvant radiotherapy.

Score	Axilla	Inferior fold
1	3 (2.4%)	24 (19.2%)
2	30 (24.0%)	32 (25.6%)
3	29 (23.2%)	12 (9.6%)
4	22 (17.6%)	20 (16.0%)
5	38 (30.4%)	33 (26.4%)
6	3 (2.4%)	4 (3.2%)

**Table 3:** Chi-square tests for skin toxicity (linear-to-linear correlation).

		Axilla (*N*)							Inferior fold (*N*)						
		1	2	3	4	5	6	(*χ* ^2^ value) *p* value	1	2	3	4	5	6	(*χ* ^2^ value) *p* value
Age	50≤	3	19	21	13	33	3	(2.908)	15	21	9	14	29	4	(6.355)
	50>	0	11	8	9	5	0	0.088^*∗∗*^	9	11	3	6	4	0	0.012^*∗*^

Laterality	Right	2	18	12	11	23	1	(0.000)	15	10	10	12	19	1	(0.143)
	Left	1	12	17	11	15	2	0.994	9	22	2	8	14	3	0.705

T-stage	is, 1	3	20	23	17	26	2	(0.179)	14	25	12	15	23	2	(0.013)
	2	0	10	6	5	12	1	0.673	10	7	0	5	10	2	0.910

N-stage	0	3	24	25	20	31	3	(0.024)	19	29	11	16	28	3	(0.039)
	1	0	6	4	2	7	0	0.877	5	3	1	4	5	1	0.843

Axillary dissection	No, SLND^†^	2	22	20	16	27	2	(0.007)	17	24	10	12	22	4	(0.122)
	ALND^‡^	1	8	9	6	11	1	0.932	7	8	2	8	11	0	0.726

Chemotherapy	No	3	12	9	11	12	0	(1.961)	12	12	4	7	11	1	(1.564)
	Yes	0	18	20	11	26	3	0.161	12	20	8	13	22	3	0.211

Hormonal therapy	No	0	9	6	4	10	3	(0.872)	6	6	4	5	8	3	(0.908)
	Yes	3	21	23	18	28	0	0.350	18	26	8	15	25	1	0.341

^†^SLND: sentinel lymph node dissection; ^‡^ALND: axillary lymph node dissection; ^*∗*^parameter with *p* < 0.05; ^*∗∗*^parameter with 0.05 ≤ *p* < 0.1.

**Table 4:** Pearson's correlation analyses for skin toxicity.

	Axilla		Inferior fold	
	*r*	*p* value	*r*	*p* value
Age (years)	0.057	0.530	0.120	0.181
Body mass index (kg/m^2^)	0.301	0.001^*∗*^	0.252	0.005^*∗*^
Breast height (cm)	0.414	<0.001^*∗*^	0.375	<0.001^*∗*^
Breast separation (cm)	0.272	0.002^*∗*^	0.215	0.016^*∗*^
*V*_100_ (%)^†^	0.404	<0.001^*∗*^	0.381	<0.001^*∗*^
Calculated dose on self-area/prescribed dose (%)	0.319	<0.001^*∗*^	0.110	0.220
Calculated dose on inner half/prescribed dose (%)	0.114	0.205	0.160	0.074^*∗∗*^
*Y*-field size (cm)	0.130	0.173	0.105	0.242
Distance from humerus head to upper border (cm)	0.135	0.142	0.037	0.691
Ratio of lateral to medial separation on upper border	0.008	0.923	0.000	0.998
Length of axilla bulging (cm)	0.011	0.907	0.040	0.658

^†^ *V*_*X*_: a covered volume by the *X*% of prescribed dose; ^*∗*^parameter with *p* < 0.05; ^*∗∗*^parameter with 0.05 ≤ *p* < 0.1.

## References

[1] Clarke M., Collins R., Darby S. (2005). Effects of radiotherapy and of differences in the extent of surgery for early breast cancer on local recurrence and 15-year survival: an overview of the randomised trials. *The Lancet*.

[2] Donovan E., Bleakley N., Denholm E. (2007). Randomised trial of standard 2D radiotherapy (RT) versus intensity modulated radiotherapy (IMRT) in patients prescribed breast radiotherapy. *Radiotherapy and Oncology*.

[3] Pignol J.-P., Olivotto I., Rakovitch E. (2008). A multicenter randomized trial of breast intensity-modulated radiation therapy to reduce acute radiation dermatitis. *Journal of Clinical Oncology*.

[4] Schnur J. B., Ouellette S. C., Dilorenzo T. A., Green S., Montgomery G. H. (2011). A qualitative analysis of acute skin toxicity among breast cancer radiotherapy patients. *Psycho-Oncology*.

[5] Schnur J. B., Zivin J. G., Mattson D. M. K. (2012). Acute skin toxicity-related, out-of-pocket expenses in patients with breast cancer treated with external beam radiotherapy: A Descriptive, Exploratory Study. *Supportive Care in Cancer*.

[6] Yoshida K., Yamazaki H., Takenaka T. (2010). Objective assessment of dermatitis following post-operative radiotherapy in patients with breast cancer treated with breast-conserving treatment. *Strahlentherapie und Onkologie*.

[7] Kraus-Tiefenbacher U., Sfintizky A., Welzel G. (2012). Factors of influence on acute skin toxicity of breast cancer patients treated with standard three-dimensional conformal radiotherapy (3D-CRT) after breast conserving surgery (BCS). *Radiation Oncology*.

[8] De Langhe S., Mulliez T., Veldeman L. (2014). Factors modifying the risk for developing acute skin toxicity after whole-breast intensity modulated radiotherapy. *BMC Cancer*.

[9] Wright J. L., Takita C., Reis I. M., Zhao W., Lee E., Hu J. J. (2014). Racial variations in radiation-induced skin toxicity severity: data from a prospective cohort receiving postmastectomy radiation. *International Journal of Radiation Oncology Biology Physics*.

[10] Blanchecotte J., Ruffier-Loubière A., Reynaud-Bougnoux A., Barillot I. (2015). Acute skin toxicity in breast intensity modulated radiotherapy using field in field technique. *Cancer/Radiothérapie*.

[11] Zhou L., Xia J., Li H., Dai J., Hu Y. (2010). Association of XRCC1 variants with acute skin reaction after radiotherapy in breast cancer patients. *Cancer Biotherapy and Radiopharmaceuticals*.

[12] Falvo E., Strigari L., Citro G. (2011). Dose and polymorphic genes xrcc1, xrcc3, gst play a role in the risk of developing erythema in breast cancer patients following single shot partial breast irradiation after conservative surgery. *BMC Cancer*.

[13] Tanteles G. A., Murray R. J. S., Mills J. (2012). Variation in telangiectasia predisposing genes is associated with overall radiation toxicity. *International Journal of Radiation Oncology Biology Physics*.

[14] Terrazzino S., La Mattina P., Masini L. (2012). Common variants of eNOS and XRCC1 genes may predict acute skin toxicity in breast cancer patients receiving radiotherapy after breast conserving surgery. *Radiotherapy and Oncology*.

[15] Park H., Choi D. H., Noh J. M. (2014). Acute skin toxicity in Korean breast cancer patients carrying BRCA mutations. *International Journal of Radiation Biology*.

[16] Bentzen S. M., Thames H. D., Overgaard M. (1989). Latent-time estimation for late cutaneous and subcutaneous radiation reactions in a single-follow-up clinical study. *Radiotherapy and Oncology*.

[17] Di Franco R., Sammarco E., Calvanese M. G. (2013). Preventing the acute skin side effects in patients treated with radiotherapy for breast cancer: the use of corneometry in order to evaluate the protective effect of moisturizing creams. *Radiation Oncology*.

[18] Ulff E., Maroti M., Serup J., Falkmer U. (2013). A potent steroid cream is superior to emollients in reducing acute radiation dermatitis in breast cancer patients treated with adjuvant radiotherapy. A randomised study of betamethasone versus two moisturizing creams. *Radiotherapy and Oncology*.

[19] Mukesh M. B., Qian W., Wilkinson J. S. (2014). Patient reported outcome measures (PROMs) following forward planned field-in field IMRT: results from the Cambridge Breast IMRT trial. *Radiotherapy and Oncology*.

[20] Chen M.-F., Chen W.-C., Lai C.-H., Hung C.-H., Liu K.-C., Cheng Y.-H. (2010). Predictive factors of radiation-induced skin toxicity in breast cancer patients. *BMC Cancer*.

[21] Mellon E. A., Sreeraman R., Gebhardt B. J., Mierzejewski A., Correa C. R. (2014). Impact of radiation treatment parameters and adjuvant systemic therapy on cosmetic outcomes after accelerated partial breast irradiation using 3-dimensional conformal radiation therapy technique. *Practical Radiation Oncology*.

[22] Landoni V., Giordano C., Marsella A. (2013). Evidence from a breast cancer hypofractionated schedule: late skin toxicity assessed by ultrasound. *Journal of Experimental and Clinical Cancer Research*.

